# Prognostic Value of Molecular Intratumor Heterogeneity in Primary Oral Cancer and Its Lymph Node Metastases Assessed by Mass Spectrometry Imaging

**DOI:** 10.3390/molecules27175458

**Published:** 2022-08-25

**Authors:** Agata Kurczyk, Marta Gawin, Piotr Paul, Ewa Chmielik, Tomasz Rutkowski, Monika Pietrowska, Piotr Widłak

**Affiliations:** 1 Maria Skłodowska-Curie National Research Institute of Oncology, Gliwice Branch, 44-102 Gliwice, Poland; 2 Clinical Research Support Centre, Medical University of Gdańsk, 80-210 Gdańsk, Poland

**Keywords:** cancer prognosis, head and neck squamous cell carcinoma, intratumor heterogeneity, long-term outcome, mass spectrometry imaging, tumor microenvironment

## Abstract

Different aspects of intra-tumor heterogeneity (ITH), which are associated with the development of cancer and its response to treatment, have postulated prognostic value. Here we searched for potential association between phenotypic ITH analyzed by mass spectrometry imaging (MSI) and prognosis of head and neck cancer. The study involved tissue specimens resected from 77 patients with locally advanced oral squamous cell carcinoma, including 37 patients where matched samples of primary tumor and synchronous lymph node metastases were analyzed. A 3-year follow-up was available for all patients which enabled their separation into two groups: with no evidence of disease (NED, *n* = 41) and with progressive disease (PD, *n* = 36). After on-tissue trypsin digestion, peptide maps of all cancer regions were segmented using an unsupervised approach to reveal their intrinsic heterogeneity. We found that intra-tumor similarity of spectra was higher in the PD group and diversity of clusters identified during image segmentation was higher in the NED group, which indicated a higher level of ITH in patients with more favorable outcomes. Signature of molecular components that correlated with long-term outcomes could be associated with proteins involved in the immune functions. Furthermore, a positive correlation between ITH and histopathological lymphocytic host response was observed. Hence, we proposed that a higher level of ITH revealed by MSI in cancers with a better prognosis could reflect the presence of heterotypic components of tumor microenvironment such as infiltrating immune cells enhancing the response to the treatment.

## 1. Introduction

Solid tumors represent a mixture of coexisting clones of cancer cells with different genetic and phenotypic features, which is called intratumor heterogeneity (ITH). Genetic ITH develops during the evolution of a tumor as a consequence of genetic alterations (mutations), which are usually (sub)clonal events. Genetic ITH is potentially reflected in a plethora of phenotypic features, including cellular morphology, gene expression, metabolism, proliferation, motility, etc. Moreover, an important component of molecular ITH results from phenotypic plasticity induced by interactions between cancer cells and different local microenvironments as well as differentiation of cancer stem cells (CSC) or epidermal to mesenchymal transition (EMT). Furthermore, differences between cancer clones in primary tumor and its metastatic outgrowth, both to lymph nodes and distant organs, represent a functionally important aspect of ITH [1,2,3]. In addition to the heterogeneity of (sub)clones of actual cancer cells, tumor heterogeneity is further increased by the presence of heterotypic elements, including immune cells, microvasculature, and connective tissues [3,4,5]. It is generally assumed that ITH facilitates the process of tumor progression and affects the effectiveness of an anti-cancer treatment due to the selection of resistant sub-clones initially present in the tumor or induced upon a treatment. It has been proposed that a high level of genetic ITH is prognostic of the worse outcome since a high level of heterogeneity is generally associated with poorer survival across diverse types of cancers [6,7,8]. On the other hand, the presence of a heterotypic component of tumor exemplified by infiltrating immune cells could be associated with a favorable outcome, which was documented in breast cancers [9,10]. Despite the fundamental relevance of ITH for cancer progression and response to treatment, its clinical implications remain insufficiently defined, partly due to the limitations of analytical tools that allow addressing actual genetic or molecular (phenotypic) heterogeneity in relevant clinical material.

Two “omics” approaches are currently used to study the molecular ITH in solid cancers. One of them is spatially resolved transcriptomics which is based on in-tissue single-cell sequencing, nominated “Method of the Year 2020” [11]), that, however, is limited to nucleic acids. The second approach, mass spectrometry imaging (MSI), addresses a wide range of molecules including proteins, lipids, metabolites, or drugs. MSI combines the analytical power of mass spectrometry with the ability to register spectra for individual positions (pixels) across the surface of tissues, which reveals molecular information annotated spatially with morphological pictures [12,13]. Therefore, MSI has been widely used in cancer research within the last decade, including several papers addressing tumor heterogeneity [14,15,16]. However, one should emphasize that the available papers addressing tumor heterogeneity had a rather methodological character and focused on the development and optimization of tools used for data analysis [17,18,19]. Hence, there are only few reports where MSI has been employed in the analysis of the actual biological role of ITH, which showed differences between the primary tumor and metastatic spread in thyroid cancer [20] or association between ITH and the prognosis of breast cancer [21].

Cancer located in the head and neck region is the 6th most common cancer, accounting for about 600,000 new cases and 300,000 deaths a year worldwide [22]. The vast majority of these cancers are head and neck squamous cell carcinomas (HNSCC), cancers derived from the stratified squamous epithelium and located in the mucosa of the upper aerodigestive tract, including the mouth, pharynx, and larynx. The etiology of HNSCC involves exposure to tobacco and alcohol and the infection with human papillomavirus in the case of oropharynx cancers [23]. HNSCC belongs to malignancies with a relatively high mutation burden, which results in a substantial genetic ITH [24,25]. Several reports indicated the association of genetic ITH with an unfavorable prognosis of this cancer [6,7,8,23,24,26,27]. Moreover, at the phenotypic level, the association between higher heterogeneity of 18F-fluorodeoxyglucose uptake and a poorer prognosis was observed in oral cancer [28]. A limited number of cases of HNSCC located in the larynx [17] and oral cavity (including tongue) [18,29] have already been analyzed using MALDI-MSI, which revealed large molecular heterogeneity of tumor areas. Here, using the same analytical approach, we aimed to address the functional importance of molecular heterogeneity of this cancer using a larger group of patients with known long-term outcomes. A group of 77 patients treated due to squamous cell carcinoma located in the oral cavity with no evidence of disease or with locoregional relapse during a 3-year follow-up was included in the study. Molecular heterogeneity of tumors and their metastases to local lymph nodes resected during the primary treatment were analyzed by MSI and compared between subgroups of patients, which revealed an association of ITH with cancer prognosis.

## 2. Results

This retrospective analysis included surgically resected cancer tissues from 77 patients treated due to locally advanced oral squamous cell carcinoma (complete resection was assumed for all included patients). Two major groups of patients were distinguished: with no evidence of disease during at least 3-year follow-up (NED; *n* = 41) and with progressive disease manifested by locoregional relapse during 3-year follow-up (PD; *n* = 36) (Table 1). MALDI-MSI was used to generate molecular maps of tryptic peptides in sections of FFPE material (spectra were registered in the 700–3000 *m*/*z* range; 1776 spectral components were identified, which represented different peptide species with their isotope envelopes; Figure S1A, Supplementary Materials). In each specimen, cancer regions of interest (ROIs) were delineated by a pathologist in the target organ and in local lymph nodes (Figure S1B), and all spectra (molecular image pixels) from these ROIs were exported for further analyses. The size of cancer ROI in the primary tumor (T) ranged from 1757 to 17,440 pixels, and the size of cancer ROI in the lymph node metastases (N) ranged from 39 to 12,189 pixels.

**Figure 1:**
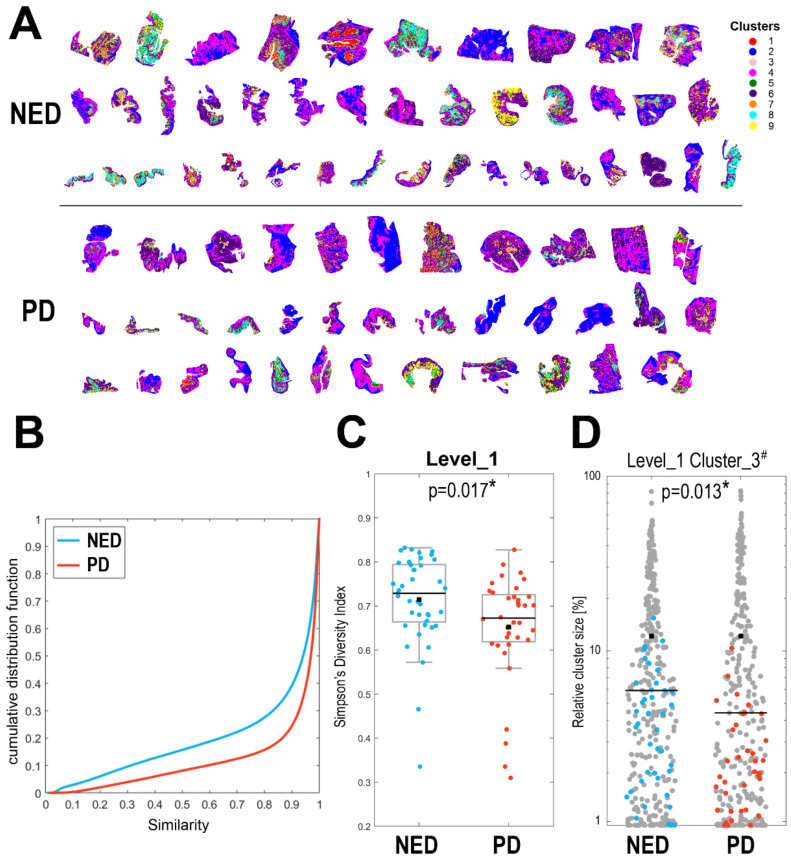
Heterogeneity of cancer ROI revealed by MALDI-MSI in oral cancer. Panel (**A**)—the distribution of 9 clusters (artificially color-coded) defined at the first level of unsupervised segmentation of cancer ROIs in 77 specimens of primary tumors analyzed together; patient groups: NED—no evidence of disease, PD—progressive disease. Panel (**B**)—the cumulative distribution function of spectra similarity index analyzed within each primary tumor (T-ROI) separately in the NED and PD groups (blue and red lines, respectively). Panel (**C**)—the Simpson’s diversity index computed for the first level of image segmentation in samples from the NED and PD groups; boxplots represent minimum, maximum, lower and upper quartile, mean (black square) and median (black line); each dot represents one T-ROI. Panel (**D**)—the relative contribution [%] of Cluster_3^#^ in the T-ROI of samples from the NED and PD groups (the selected cluster is marked in blue/red, all other clusters are shown in grey). Shown is the *p*-value of differences between NED and PD (*p* < 0.05 is marked with asterisks).

To search for the molecular ITH within primary tumors, spectra from all 77 T-ROIs (608,424 spectra together) were clustered using an unsupervised procedure based on the DivIK algorithm [18,30]. The first four steps (levels) of such image segmentation generated 9, 38, 181, and 1005 clusters, respectively; Figure 1A illustrates the distribution of 9 clusters generated in all 77 samples at the Level_1 of the procedure. Clusters generated during image segmentation correlated with histological structures observed in serial sections, which is exemplified in Figure S1C. We first assessed the overall similarity of spectra within each T-ROI; graphs illustrating the cumulative distribution function of the similarity index calculated for each patient are presented in Figure S2. Figure 1B represents the aggregated value of this function in both groups of patients. We observed a higher value of the similarity index in the PD group as compared to the NED group (median value of 0.969 and 0.941, respectively; see details in Table S2), which suggested a higher level of heterogeneity in the latter group.

Then, we looked at the number of clusters identified by the unsupervised procedure in each T-ROI. In general, starting from the Level_3 of image segmentation a positive correlation between the size of a ROI (i.e., the number of spectra) and the number of clusters was observed (Figure S3A); however, in both groups of patients the sizes of ROIs were similar (Figure S3B), thus the comparison based on the number of clusters was credible. We found that the numbers of clusters identified in the T-ROIs were generally higher in the NED group (Figure S4A), yet the differences did not reach the level of statistical significance (*p* > 0.05). Therefore, we applied the Simpson’s diversity index, which took into consideration both the number and the size of clusters (this index estimates the probability that two pixels belong to different clusters—a higher index value reflects a lower probability of co-clustering resulting from higher heterogeneity). We found that this index estimated in the T-ROIs was significantly higher in the NED group (Figure S4B); Figure 1C compares the values of Simpson’s index estimated at the Level_1 of image segmentation in cancer specimens between both groups of patients (*p* = 0.017). The presented results confirmed collectively a higher level of intra-ROI heterogeneity in specimens of primary tumor in the NED group compared to the PD group.

For comparison, we also analyzed differences in molecular ITH among tumors located in different parts of the oral cavity and among tumors from patients with different cancer stages at the time of initial diagnosis (independent of the long-term outcomes). We found that the diversity index in primary tumors was similar in patients with different tumor size T (*p* = 0.624), local lymph node status N (*p* = 0.122), and clinical cancer stage (*p* = 0.445); Figure S5A–C. Moreover, differences in heterogeneity were not observed between tumors with different locations in the oral cavity (*p* = 0.132; Figure S5C). Therefore, clinical cancer status (stage and location) was not associated with the heterogeneity revealed by MALDI-MSI, which suggested an independent prognostic value of this parameter. To compare the structure of clusters in the NED and PD groups, we analyzed the relative contribution of different clusters in each T-ROI. We found a few clusters that had a significantly higher contribution in the T-ROI of the NED samples than the PD samples, which is exemplified by Cluster_3^#^ detected at the Level_1 of image segmentation (Figure 1D).

**Figure 2:**
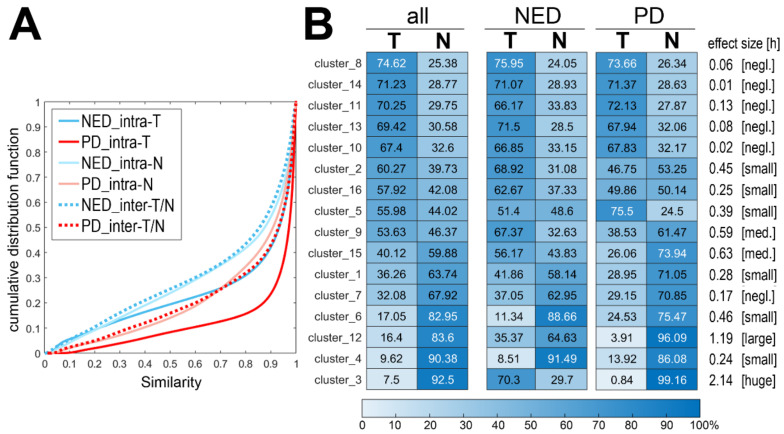
Differences in molecular profiles of primary tumors and lymph node metastases. Panel (**A**)—the cumulative distribution function of the spectra similarity index analyzed either within each type of ROI (intra-T and intra-N) or between types of cancer ROI (inter-T/N) separately in the NED and PD groups (blue and red lines, respectively). Panel (**B**)—the relative contribution of each cluster defined at the second level of image segmentation in primary tumor (T) and lymph node metastases (N). Shown are values obtained for all 37 T/N pairs, 17 T/N pairs in the NED group, and 20 T/N pairs in the PD group; the average relative participation of T-ROI and N-ROI [%] in a given cluster is color-coded in the heat map. Significance of differences between groups was assessed using the clusterization [h] effect size (negl.—negligible, med.—medium).

To further analyze the potential association between molecular ITH revealed by MALDI-MSI and patients’ outcomes, the differences between primary tumors and their metastases to local lymph nodes were compared between the NED and PD groups. In this part, we have analyzed the material of 37 patients for whom paired specimens of the tumor and cancer spread to lymph nodes resected during the primary treatment were available, which included 17 patients from the NED group and 20 patients from the PD group. Spectra from all T-ROIs and N-ROIs (480,455 spectra altogether) were clustered using the unsupervised procedure as described above, which resulted in 3, 16, 76, and 373 clusters at the first four levels of segmentation (Figure S6 illustrates the distribution of 16 clusters generated in all specimens at the Level_2 of the procedure). First, we assessed the overall similarity of spectra within (“intra”) T-ROI and N-ROI as well as the similarity of spectra between (“inter”) paired T-ROI and N-ROI for each patient, as described previously for thyroid cancer [20] (corresponding cumulative distribution functions of the similarity index calculated for each patient are presented in Figure S7). We found that intra-ROI similarity was lower in the case of lymph node metastases (N) when compared to primary tumors (T). In both cases, however, similarity indexes were higher in the PD group compared to the NED group (aggregated medium values of 0.973, 0.933, 0.909, and 0.876 for PD-T, NED-T, PD-N, and NED-N, respectively). Moreover, the T/N inter-ROI similarity was lower in the NED group compared to the PD group (aggregated medium value of 0.855 and 0.931, respectively), which is illustrated in Figure 2A (and Table S1). Furthermore, we analyzed the relative contribution of different clusters in both types of cancer ROIs. The heat map illustrated in Figure 2B shows the relative participation of T-ROI and N-ROI in each of 16 clusters identified at the Level_2 of image segmentation (noteworthy, the sizes of the analyzed T-ROIs and N-ROIs were comparable—the average ratio between matched T-ROI and N-ROI was 1.4 (T/N)). We found groups of clusters that predominated in either T-ROI or N-ROI, and clusters with equal distribution between both types of cancer ROIs; the spatial distribution of clusters typical of T-ROI (exemplified by Cluster_8) and typical of N-ROI (exemplified by Cluster_4) is presented in Figure S8. Then, we searched for putative differences in the relative contribution of T-ROI and N-ROI between both groups of patients. This revealed that in four clusters (Cluster_3, 9, 12, and 15) significant differences in the T/N distribution were observed between the NED and PD groups. In each case participation of a cluster characteristic for N-ROI in the PD group, increased in the T-ROI in the NED group (Figure 2B).

**Figure 3:**
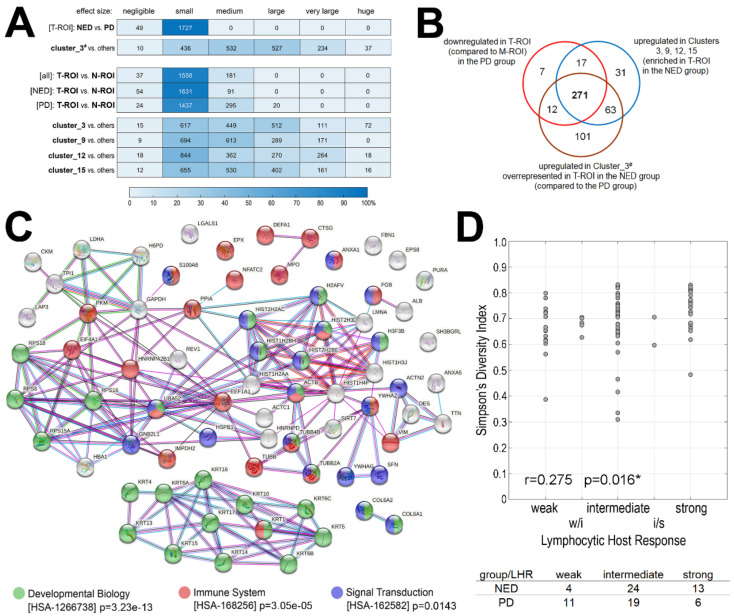
Components that differentiated samples of patients with a different outcome. Panel (**A**)—the number of spectral components with a different Cohen’s [d] effect size between different pairwise compared tissue regions; the relative number of components [%] with increased significance of differences is color-coded in a heat map. Panel (**B**)—the network of interactions between 74 proteins, the tryptic fragments of which were putatively identified as spectral components downregulated in T-ROI (compared to N-ROI) in the PD group specifically; interaction between proteins and over-represented Reactome pathway functions associated with these proteins according to an analysis using the STRING toolbox [31] (color-coded are proteins associated with 3 most numerous overrepresented pathways, shown is corrected p-value of pathway over-representation). Panel (**C**)—Venn diagram showing the overlap between MSI components downregulated in T-ROI (vs. N-ROI) in PD group, upregulated in clusters relatively enriched in T-ROI (vs. N-ROI) in NED group, and upregulated in a cluster enriched in T-ROI in NED group. Panel (**D**)—the correlation between ITH (the Simpson’s diversity index computed for the first level of image segmentation) and Lymphocytic Host Response (LHR) [32] assessed by histopathology analysis in corresponding specimens (w/i—weak/intermediate, i/s—intermediate/strong); number of samples with different LHR in the NED and PD groups (bottom; “w/i” and “i/s” were combined with “intermediate”); * indicates statistical significance (*p* < 0.05).

To characterize molecular features of tissue samples collected during primary surgery from patients with different outcomes, spectral components that showed significantly different abundance in different types and regions of specimens were detected (the Cohen’s [d] effect size was used to determine the significance of differences; Tables S2 and S3). Furthermore, the hypothetical identity of MSI components was established by attributing masses (*m*/*z* values) of spectral components (i.e., tryptic peptides) to measured masses of peptides identified by LC-MALDI-MS/MS in lysates from cancer tissue (Table S4). This enabled identification of proteins matched to 557 MSI components (however, this type of annotation is not unique due to relatively low resolution of MALDI-ToF MSI and more than one peptide could be matched to certain MSI components). When T-ROIs from NED and PD samples were compared directly, no statistically significant differences in the abundance of MSI components were noted (Table S2). However, when we looked at the composition of Cluster_3^#^ (Level_1 of the clusterization of all 77 T-ROIs) overrepresented in the NED group (Figure 1D), 447 MSI components were found that were significantly upregulated (at least medium effect size) in this cluster compared to other areas of T-ROI (Figure 3A and Table S2). The most significantly upregulated MSI components (at least large effect size) were matched to fragments of identified proteins, which revealed 87 proteins involved in different (Reactome) pathways (Table S4). Noteworthy, the most numerous subsets of proteins putatively matching MSI components upregulated in Cluster_3^#^ overrepresented in the NED samples were those involved in “Developmental Biology”, “Immune System”, and “Signal Transduction” (Figure S9).

Next, we searched for MSI components that showed different relative abundance in cancer ROIs present in the primary tumor and in lymph node metastases. We found that more MSI components showed significantly different abundance (medium or large effect size) between T-ROI and N-ROI in the PD group compared to the NED group: 315 and 91 components, respectively (Figure 3A, Table S3). Noteworthy, the majority of differentiating MSI components specific to the PD group (i.e., not differentiating T-ROI vs. N-ROI in the NED group) were relatively downregulated in T-ROI (compared to N-ROI). MSI components downregulated in T-ROI of the PD group (307 components) were matched to fragments of identified proteins, which revealed 74 proteins (Table S4). The most numerous subsets of matching proteins were those involved in “Developmental Biology”, “Immune System”, and “Signal Transduction” (Figure 3B). Finally, we looked at MSI components upregulated (at least medium effect size) in clusters with increased contribution to T-ROI in the NED group compared to the PD group, i.e., Clusters_3, 9, 12, and 15 (Level_2 of the clusterization of 37 T/N-ROIs; Figure 2B) and found 382 MSI components that were upregulated in at least 3 out of 4 clusters. Noteworthy, 288 of them were also specifically downregulated in T-ROI in the PD group. Moreover, 283 MSI components downregulated in the T-ROI in the PD group (compared to corresponding N-ROI) were also upregulated in Cluster_3^#^ overrepresented in T-ROI in the NED group (Figure 3C). We concluded that although a direct comparison of T-ROI between samples of NED and PD groups did not reveal a prognostic signature of MSI components, indirect approaches enabled finding features associated with different outcomes. Importantly, sets of features that were relatively upregulated in primary tumors from NED samples (i.e., components upregulated in Cluster_3^#^ and components upregulated in four clusters that increased contribution to T-ROI in NED) and relatively downregulated in primary tumors from PD samples (i.e., downregulated in T-ROI compared to N-ROI specifically in PD) largely overlapped. It is noteworthy that a large set of features characteristic of primary tumors in the NED group included MSI components putatively matching proteins associated with immune functions.

To address directly the correlation between ITH and the presence of immune cells, we assessed the so-called Lymphocytic Host Response (LHR) which is based on the pattern of immune cell infiltration at the tumor/host interface. This parameter is an element of the histological risk assessment model proposed for oral cancers by Brandwein-Gensler and coworkers [32]. According to this model, none/weak infiltration was associated with a higher risk of recurrence and a strong response (with dense/continuous pattern of infiltration) was associated with increased probability of local disease-free and overall survival. Here we observed a statistically significant over-representation of strong LHR in the NED group and over-representation of weak LHR in the PD group (*p* = 0.041; Figure 3D, bottom). More importantly, we found a positive correlation between the LHR and Simpson’s Diversity Index (*p* = 0.016; Figure 3D). Moreover, clusters of cytotoxic T cells and helper T cells were observed at borders of tumor areas in specimens with a high ITH (Figure S10). All these observations strengthen our conclusion on the mechanistic association between phenotypic ITH and the presence of infiltrating immune cells.

## 3. Discussion

Head and neck cancer, exemplified by squamous cell carcinoma located in the oral cavity, represents malignancy with a substantial level of intratumor heterogeneity. This heterogeneity was observed at the genetic level, both concerning single nucleotide variations (SNV) and copy number variations (CNV) [24,33]. Phenotypic heterogeneity of oral cancer was noted at the level of gene expression [34], protein patterns [17,18,29], and metabolic features [28]. Moreover, variability of heterotypic components was observed at the level of transcription profiles of tumor-infiltrating lymphocytes [35]. Several studies revealed the association of intratumor heterogeneity with the prognosis of patients with oral cancer. An increased level of genetic heterogeneity of oral cancer, either SNV [26,27] or CNV [8] was associated with worse outcomes. Interestingly, genetic heterogeneity of morphologically normal mucosa adjacent to oral cancer was also associated with the patient’s prognosis [36]. At the phenotype level, increased heterogeneity of the 18F-FDG uptake in oral cancer was also associated with a worse prognosis [28]. Here we addressed the association of long-term outcomes of patients treated due to oral cancer with the heterogeneity of protein profiles assessed by MSI in cancer specimens resected during the primary treatment. Unexpectedly, we found that a higher level of molecular intratumor heterogeneity was correlated with favorable outcomes. We observed that the level of similarity between image pixels within cancer ROI was lower, as well as the number and diversity of clusters generated during the unsupervised segmentation of molecular images were higher in cancer specimens collected during the initial surgery from patients with no evidence of disease after at least a 3-year follow-up. On the other hand, in specimens collected from patients who suffered a loco-regional relapse, the similarity of image pixels was higher although the diversity of image clusters was lower. Hence, considering this “counter-intuitive” picture, it is important to understand the nature of intratumor heterogeneity that could be addressed using the applied MALDI-MSI approach.

An important aspect of intratumor heterogeneity is the difference between the primary cancer and its synchronous metastases. In the case of head and neck cancer, particularly oral cancer, distant metastases are relatively rare compared to other malignancies and spread to local or regional lymph nodes predominates [37]. Moreover, genetic differences between primary oral tumors and metastatic sites are relatively low [38]. Here we compared molecular profiles of primary tumors and lymph node metastases and found that the level of similarity index between matched primary tumor and metastasis was lower than the similarity within the primary site. However, the similarity between primary tumor and lymph node metastases observed in oral cancer was markedly higher than in papillary thyroid cancer addressed by MSI in the previous study (median similarity 0.89 and 0.61, respectively). Noteworthy, major differences between primary thyroid tumors and lymph node metastases included upregulation of proteins associated with immune function in metastases [20]. Here we observed relatively fewer differences between primary oral cancer and its lymph node metastases (compared to thyroid cancer). Nevertheless, molecular differences between primary tumor and metastases were more intense in the cases of patients with worse outcomes, which implicated more similar protein profiles between primary tumor and lymph node metastases in patients with better outcomes. Importantly, molecular components relatively downregulated in primary tumors (compared to lymph node metastases) of patients with worse prognoses were putatively associated with proteins with immune-related functions, which suggested a relatively higher level of such proteins in primary tumors of patients with better prognoses.

Direct comparison of molecular profiles of primary tumors did not reveal components with significantly different abundance in patients with different outcomes. However, a few indirect MSI-based approaches proposed in this report indicated coherently that an increased abundance of components putatively associated with immune-related proteins was characteristic of primary tumors in patients with favorable outcomes, which turned our attention to the immune component of the tumor microenvironment. One should be aware that due to the relatively low lateral resolution of MALDI-MSI maps analyzed in this study, a single image pixel corresponds to a cluster of several cells. Hence, the presence of heterotypic components of the tumor microenvironment, e.g., clusters of cells with separate phenotypes and functions, is a putative target of MALDI-MSI. We have previously found that a higher level of intratumor heterogeneity addressed by MALDI-MSI was associated with better outcomes in patients with HER2-positive breast cancers. Moreover, increased heterogeneity observed in that study was associated with the presence of tumor-infiltrating lymphocytes, which prompted us to hypothesize that a more favorable prognosis of breast cancer patients who had a higher level of ITH revealed by MALDI-MSI was associated with a higher level of immune cells enhancing the response to treatment [21]. We found here that continuous/dense pattern of lymphocyte infiltration at the tumor/host interface correlated with a higher level of phenotypic ITH, which suggested similar mechanisms in patients with locally advanced oral cancer. Thus, a general importance of this phenomenon could be assumed. Nevertheless, our observation is in line with previous studies which showed that the level/pattern of tumor-infiltrating lymphocytes was associated with a better prognosis for patients with head and neck cancer [32,39,40].

In conclusion, we found that phenotypic heterogeneity revealed by mass spectrometry imaging within tumor tissue resected from patients with locally advanced oral cancer was higher in patients with favorable outcomes. On the other hand, neither clinical cancer stage nor cancer localization was associated with the observed intratumor heterogeneity, which suggested an independent prognostic value of this parameter. Moreover, the level of ITH was associated with histopathological pattern of infiltrating lymphocytes and the signature of molecular components that correlated with long-term outcomes could be associated with proteins involved in the immune functions. Hence, we hypothesized that higher intratumor heterogeneity observed by mass spectrometry imaging in cancers with a better prognosis could reflect the presence of heterotypic components such as infiltrating immune cells enhancing the response to the treatment.

## 4. Materials and Methods

### 4.1. Clinical Material

Samples of 77 patients (56 males and 21 females, aged from 29 to 81 years, median 58) with locally advanced oral squamous cell carcinomas were included in the study. The majority of primary tumors were located on the floor of the mouth (53%) and tongue (34%); 36 patients had no metastases in local lymph nodes (N0), and in 41 patients cancer cells were detected in lymph nodes collected during lymphadenectomy (N1–N2). Radical surgery was the primary treatment for all patients, which was followed by adjuvant radiotherapy (72%) or radio-chemotherapy based on cisplatin (21%). No evidence of diseases was observed in 41 patients during at least a 3-year follow-up (NED group; follow-up from 36 to 128 months, median 78 months). Locoregional relapse was diagnosed in 36 patients during the same follow-up (progressive disease—PD group; relapse was diagnosed from 1 to 44 months after the end of therapy, median 8 months). Table 1 presents more detailed information about the compared groups. Postoperative tissue collected during surgery was stored as formalin-fixed paraffin-embedded material (the surgery was performed in the years 2008–2016). The tissue material was re-inspected and verified by experienced pathologists before the study.

### 4.2. Reagents

The following solvents were used for processing of tissue sections on ITO glass slides: xylene (mixture of isomers) pure per analysis (POCh, Gliwice, Poland), ethanol HPLC grade (POCh, Gliwice, Poland), demineralized water from a Simplicity UV system equipped with an LC-Pak Polisher filter (both from Millipore SAS, Molsheim, France). Sequencing Grade Modified Trypsin from Promega (Madison, WI, USA) was used as a proteolytic enzyme. Ammonium bicarbonate BioUltra was purchased from Sigma Aldrich (St. Louis, MO, USA), acetonitrile (ACN), methanol (MeOH) and trifluoroacetic acid (TFA)—all HiPerSolv CHROMANORM for LC-MS grade—were obtained from VWR Chemicals (Radnor, PA, USA). ITO glass slides, alpha-cyano-4-hydroxycinnamic (HCCA) and Peptide Calibration Standard II were purchased from Bruker Daltonik (Bremen, Germany). Poly-l-lysine solution (0.1% *w/v* in H_2_O) and IGEPAL^®^ CA-630 were both obtained from Sigma-Aldrich. ITO glass slides were coated with poly-l-lysine according to a procedure given in [20]. *Rapi*Gest SF Surfactant and TopTips (10–200 µL) C18 were purchased from Waters (Milford, MA, USA) and Glygen Corp. (Columbia, MD, USA), respectively.

### 4.3. FFPE Tissue Sectioning and Preparation for MALDI-MSI

All FFPE tissue blocks were sectioned using a Microm HM 304E rotary microtome (Microm International GmbH, Walldorf, Germany). Material from an individual patient (one 3 µm-thick section from a primary tumor block and one 3 µm-thick section from a metastatic lymph node—if available) were placed on the same ITO slide in duplicate, a consecutive 3 µm-thick section of both primary tumor and metastatic lymph node was placed on a standard poly-lysine glass slide for H and E staining. An additional five 10 µm-thick sections were collected in a 1.5 mL Eppendorf SafeLock tube for further LC-MALDI-MS/MS analysis of protein extract. All slides were subsequently dried for 1 h at 56 °C, and stored at room temperature until processed further. Prior to paraffin removal, ITO slides with tissue sections were heated for 30 min at 60 °C (using a Thermomixer Comfort, Eppendorf, equipped with a slide adapter) for better adherence. Next, the paraffin was removed and sections were boiled in a Decloaking Chamber (Biocare Medical, Pacheco, CA, USA) for reversal of protein crosslinking and dried. Trypsin deposition was performed using a SunCollect tissue sprayer (SunChrom GmbH, Friedrichsdorf, Germany), then a section was placed in a humid chamber containing a solution of 100 mM NH_4_HCO_3_, 5% MeOH and kept at 37 °C for 18 h. After that time a matrix solution (5 mg/mL HCCA in 50% ACN, 0.3% TFA) was deposited onto the section using SunCollect. A scheme of FFPE blocks sectioning along with all details of tissue preparation for MALDI-MSI are given in Supplementary Protocols S1–S4.

### 4.4. MALDI-MSI Measurements

MALDI-mass spectrometry imaging measurements were realized using an ultrafleXtreme MALDI-ToF mass spectrometer (Bruker Daltonik, Bremen, Germany) equipped with a smartbeam II laser operating at 1000 Hz repetition rate. Bruker Compass for FLEX series 1.4 (including flexImaging 4.1) was used for spectra acquisition and handling. The spectrometer was operated in positive reflectron mode with smartbeam parameter set: 4_large, acceleration voltage of 20 kV, and PIE of 150 ns. Spectra were recorded in 700–3000 *m*/*z* range with raster width of 100 µm; 400 spectra were collected from each position (random walk activated, 40 shots at raster spot). Sampling rate was set at 4.00 GS/sec in order to provide at least 10 points/peak for the needs of subsequent Gaussian mixture modeling. ToF analyzer was externally calibrated with Peptide Calibration Standard II (Bruker), then Statistical Peptide calibration strategy was employed (taking into consideration 6 different positions on a tissue section). Material from a single patient was measured on the same ITO slide. The whole sample set was measured in a random order.

### 4.5. Spectra Processing

The acquired MSI spectra were preprocessed by computationally performed mass channels unification, baseline subtraction, outlying spectra identification, peak alignment, TIC normalization, and peak detection using Gaussian mixture modeling (GMM) of the average spectrum; the procedure of our spectra processing pipeline was described in detail elsewhere [21,41]. Abundance of a particular component was estimated by pairwise convolution of the GMM components and individual spectra. The resulting MSI dataset consisted of 608,424 spectra acquired from 77 imaged tissue specimens. Each spectrum of the MSI dataset featured 1776 components that represented tryptic peptide species.

### 4.6. Statistical Analyses

Pairwise similarity index was calculated to assess similarities between a compared pair of spectra [42]. The similarity index was computed in two manners: within particular ROI (intra-ROI similarity; e.g., intra-tumor [intra-T], intra-metastasis [intra-N]), and between different ROIs (inter-ROIs similarity; e.g., inter-T/N) creating all possible combinations of compared spectra pairs. Populations of computed similarity values were plotted as cumulative distribution functions to visualize the intra-ROI heterogeneity and inter-ROIs differences with relevance to the NED and PD patient groups. The deglomerative divisive iK-means (DivIK) algorithm with region-driven feature selection was applied for unsupervised molecular image segmentation as described in detail elsewhere [18,21,30]. Spectra from (i) all 77 T-ROIs, and spectra from (ii) 37 patients for whom paired specimens of the T-ROI and N-ROI were available, created two distinct datasets that were clustered separately. Cohen’s [d] effect size was calculated to indicate discriminatory components between pairwise compared subsets of spectra (the [d] value was defined as the difference between the mean abundance of a component divided by the pooled standard deviation for the two compared groups of spectra) [43]. Simpson’s diversity index [44] was calculated to assess T-ROIs heterogeneity (Simpson’s index reflects the probability that two randomly selected T-ROI spectra belong to different clusters). Due to violations of the applicability assumptions of the parametric testing of statistical hypotheses, the nonparametric tests were applied to determine significance of differences in the number of clusters, Simpson’s diversity index, and cluster sizes. The Wilcoxon rank-sum test, or the Kruskal–Wallis test were applied if two or more than two groups were compared, respectively. Cohen’s [h] effect size (defined as the difference between two proportions which were transformed by arcsine function) [43] was calculated to quantify the size of the difference between proportions of T-ROIs and N-ROIs spectra of particular clusters for the NED and PD patient groups. To estimate the significance of differences in patients’ age with relevance to the NED and PD groups, the two-sample Student’s t-test was applied, since the assumptions for applicability of parametric tests were met. The Fisher’s exact test was applied to test whether clinical descriptors, e.g., tumor location, tumor size, lymph node status and cancer stage were the NED and PD group-related factors. All statistical hypotheses were tested at the 5% significance level.

### 4.7. Protein Identification by LC-MALDI-MS/MS

Protein identification was performed for protein lysates obtained from consecutive tissue sections collected during FFPE block sectioning. Five cases containing ca. 50% tumor cells were randomly selected both for primary tumor tissues and for lymph node tissues, and processed according to a modified procedure by Geoui et al. [45] as given in Supplementary Protocol S5. Purified protein extracts dissolved in 0.1% *Rapi*Gest in 50 mM NH_4_HCO_3_ were subsequently merged within a group, i.e., primary tumor tissue extracts and lymph node tissue extracts were merged separately (a volume equivalent to 10 µg protein was used for each sample), and thus obtained protein mixtures were subjected to tryptic digestion according to Supplementary Protocol S6. The final tryptic peptide content in each mixture was assessed using the tryptophan fluorescence method [46]. Three portions (technical replicates) of each mixture containing 8 µg of tryptic peptides were then analyzed using LC-MALDI-MS/MS technique as described in detail elsewhere [47]. Briefly, separation of peptides was performed with the use of Proxeon EASY-nLC nano-liquid chromatograph coupled with PROTEINEER fc II fraction collector (Bruker). Fractions collected on an MTP AnchorChip 1536 BC target plate were analyzed using ultrafleXtreme MALDI-ToF mass spectrometer operated in positive reflectron mode with ac-celeration voltage of 25 kV, PIE of 150 ns, and detection range of 700–3500 *m*/*z*. The laser was operated at 1000 Hz repetition rate with random walk activated (100 shots at raster spot). MS/MS spectra acquired from the replicates were then combined within a group and subjected to protein search using ProteinScape 3.1 platform (Bruker) and Mascot Server 2.5.1.1 (Matrix Science Ltd., London, UK). SwissProt database 2022_1 (566,996 sequences; 204,698,499 residues) was employed with the taxonomy set at homo sapiens (20,377 sequences). MS and MS/MS tolerance was set at 25 ppm and 0.5 Da, respectively. Raw LC-MALDI-MS/MS data are available in the ProteomeXchange/PRIDE repository [48,49] with the dataset identifier PXD034958. The hypothetical identity of the MSI components was established by the assignment of a component location on the *m*/*z* scale for the measured masses of tryptic peptides identified by LC-MS/MS allowing ±0.05% mass tolerance.

## Figures and Tables

**Table 1:** Clinical characteristics of patients included in the study.

Group *	NED	PD	Significance of Differences between Groups
*n*	41	36	
age (min–max (median))	29–81 (58)	41–78 (58)	*p* = 0.679
sex (male/female)	27/14	29/7	*p* = 0.201
tumor location:			*p* = 0.151
floor of mouth	25	16	
tongue	14	12	
other	2	8	
tumor size:			*p* = 0.025
T1	4	6	
T2	25	11	
T3	12	16	
T4a	-	3	
lymph node status:			*p* = 0.682
N0	22	14	
N1	10	9	
N2	9	13	
cancer stage:			*p* = 0.114
I	4	2	
II	13	5	
III	15	13	
IVA	9	16	

* NED—no evidence of disease; PD—progressive disease.

## Data Availability

The LC-MALDI-MS/MS-based proteomic data have been deposited in the ProteomeXchange Consortium via the PRIDE (https://www.ebi.ac.uk/pride, accessed on 17 June 2022) [48,49] partner repository with the dataset identifier PXD034958. The remaining data presented in this study are available on request from the corresponding author. The data are not publicly available due to privacy and ethical reasons.

## Supplementary Materials

The following supporting information can be downloaded at: https://www.mdpi.com/article/10.3390/molecules27175458/s1, Table S1: The intra-ROI and inter-ROI similarity index for MSI spectra, Table S2: Significance of differences (Cohen’s [d] effect size) for abundances of spectral components between specific T-ROIs, Table S3: Significance of differences (Cohen’s [d] effect size) for abundances of spectral components between primary cancer (T-ROI) and lymph node metastases (N-ROI), Table S4: Hypothetical identity of MSI components based on their assignment to measured masses of tryptic peptides identified by LC-MS/MS, Figure S1: Characterization of imaged tissue specimens, Figure S2: The cumulative distribution function of the spectra similarity index analyzed within the primary tumor (T-ROI) for each patient in the NED and PD groups, Figure S3: Size of cancer ROI in tissue specimens analyzed by MALDI-MSI, Figure S4: Differences in heterogeneity of cancer ROI between the NED and PD groups of patients, Figure S5: Heterogeneity of cancer ROI assessed by the Simpson’s diversity index at the first level of unsupervised image segmentation in different subgroups of patients, Figure S6: The distribution of clusters identified during the unsupervised segmentation of cancer ROI in both primary tumor (T-ROI) and synchronous lymph node metastases (N-ROI), Figure S7: The cumulative distribution function of the spectra similarity index analyzed for each patient in the NED (17 pts.) and PD (20 pts.) groups, Figure S8: The distribution of clusters identified at the second level of unsupervised segmentation of cancer ROI (both T-ROI and N-ROI) in samples of patients from the NED and PD groups, Figure S9: The network of interactions between 87 proteins the tryptic fragments of which were putatively identified as spectral components with abundances significantly upregulated in Cluster_3^#^ compared to other clusters at the first level of unsupervised image segmentation of all T-ROI areas, Figure S10: Infiltration of lymphocytes at the tumor/host interface, Supplementary Protocol S1: Preparation of tissue material for an individual patient, Supplementary Protocol S2: Paraffin removal procedure, Supplementary Protocol S3: Heat-induced protein crosslinking reversal procedure, Supplementary Protocol S4a: On-tissue trypsin digestion procedure, Supplementary Protocol S4b: SunCollect settings for trypsin and matrix deposition, Supplementary Protocol S5: Preparation of tissue lysates for LC-MALDI-MS/MS protein identification, Supplementary Protocol S6: Preparation of protein digests for LC-MALDI-MS/MS protein identification.
