# Supplementary material for: Prognostic Value of Molecular Intratumor Heterogeneity in Primary Oral Cancer and Its Lymph Node Metastases Assessed by Mass Spectrometry Imaging
Source: Molecules. 2022 Aug 25;27(17):5458. doi: 10.3390/molecules27175458 (PMC9458238; doi:10.3390/molecules27175458)
Supplement: Supplementary file 1 [file molecules-27-05458-s001.zip › Supplementary Materials/Supplementary Material - Figures & Protocols.pdf]

Prognostic value of molecular intratumor heterogeneity in primary oral cancer and its lymph node metastases assessed by mass spectrometry imaging.

Supplementary Materials

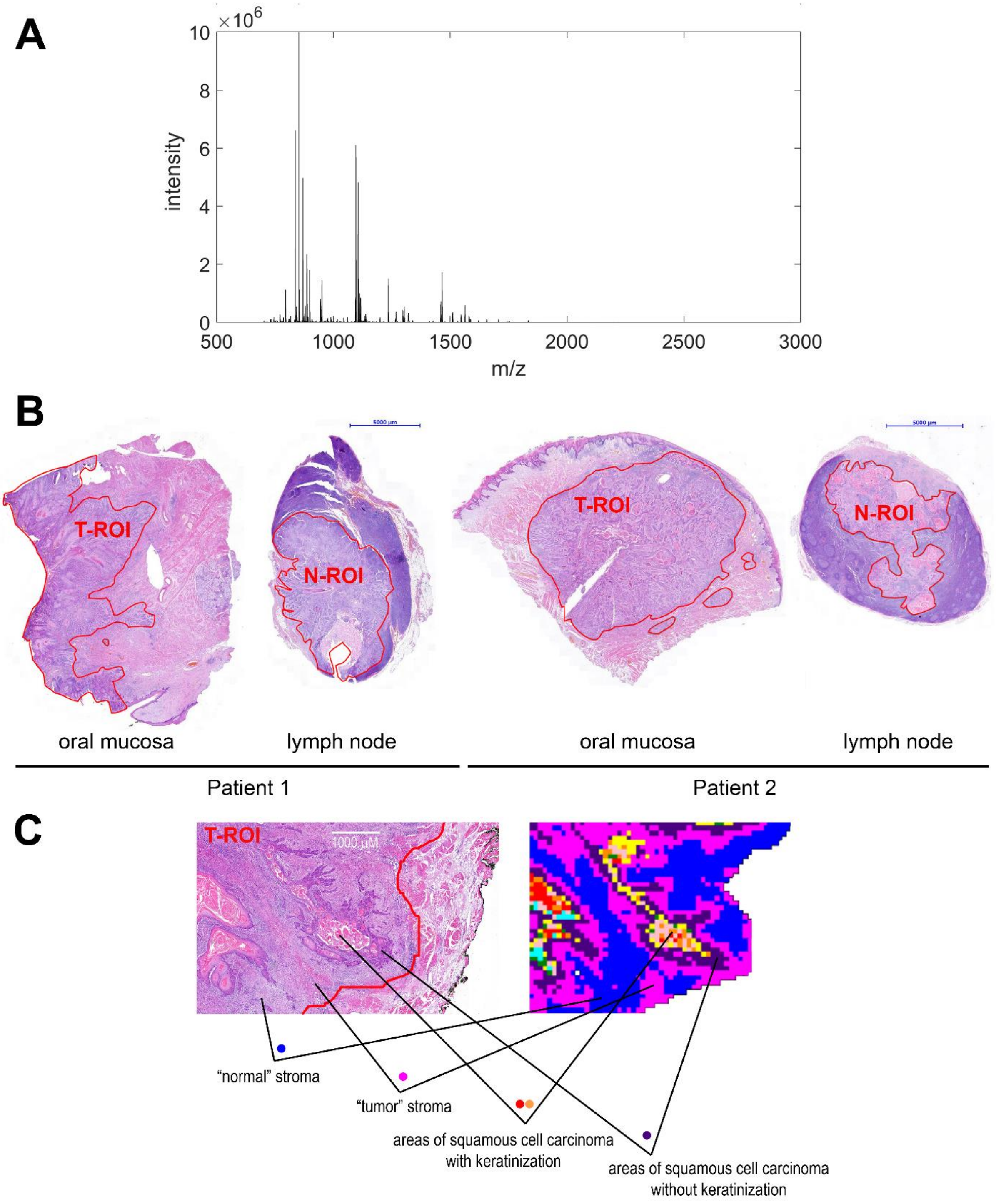

Supplementary Figure S1. Characterization of imaged tissue specimens. **Panel A** - Average MALDI-ToF mass spectrum of the cancer tissue. **Panel B** – Cancer Region of Interest (ROI) delineated in a tissue specimen, either primary cancer within oral mucosa (T-ROI) or metastases in lymph nodes (N-ROI). Examples of two pairs of tissue sections stained with hematoxylin and eosin (H&E) that were used to mark cancer ROI – tissue area within the red line; scale bar corresponds to 5,000 µm. **Panel C –** Correlation between histopathological structures visible on H&E section (left) and clusters generated by unsupervised MSI image segmentation in serial section of a tissue specimen (right).

Prognostic value of molecular intratumor heterogeneity in primary oral cancer and its lymph node metastases assessed by mass spectrometry imaging.

Supplementary Materials

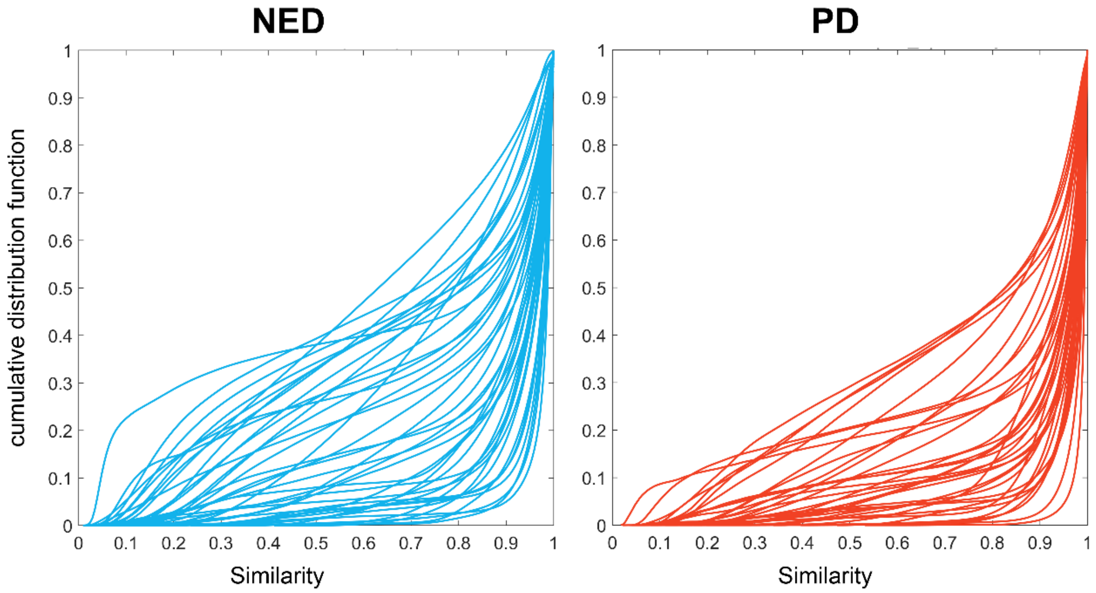

Supplementary Figure S2. The cumulative distribution function of the spectra similarity index analyzed within the primary tumor (T-ROI) for each patient in the NED and PD groups.

Prognostic value of molecular intratumor heterogeneity in primary oral cancer and its lymph node metastases assessed by mass spectrometry imaging.

Supplementary Materials

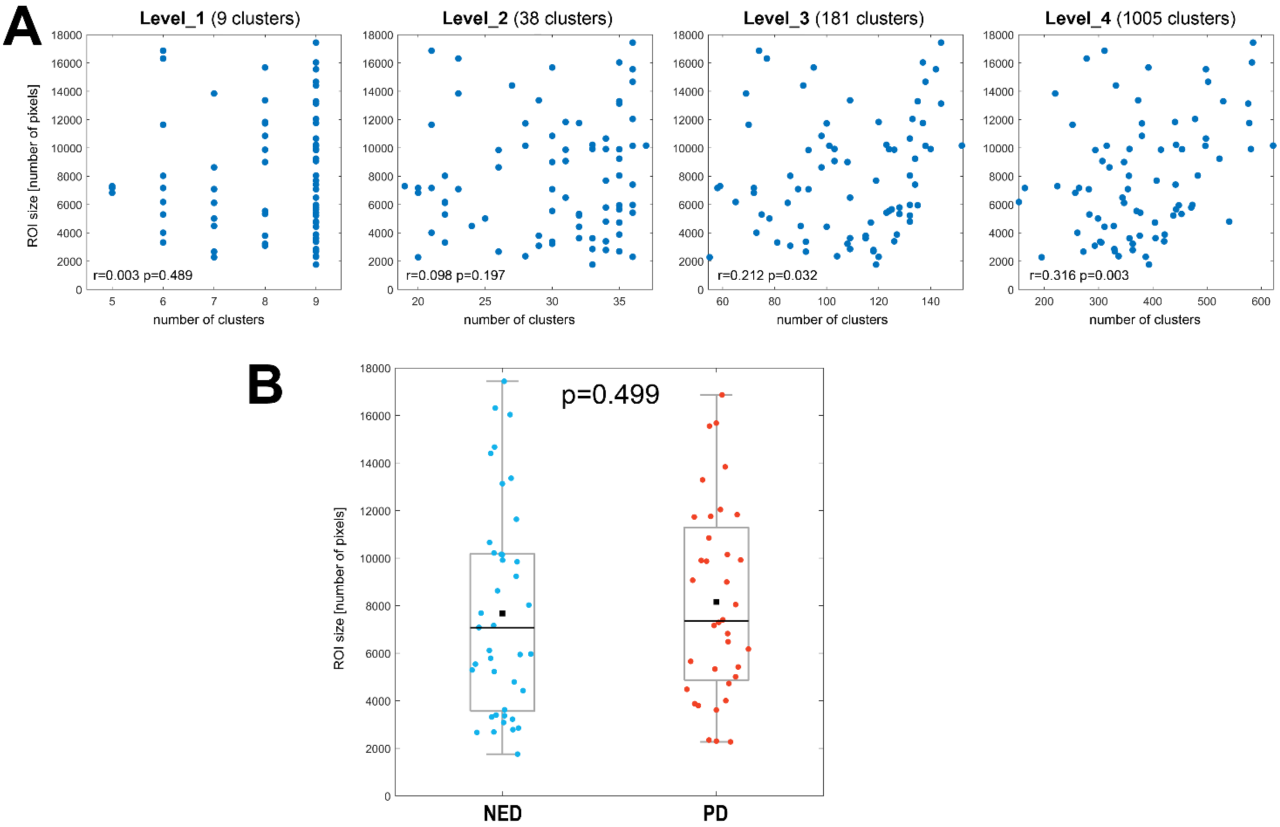

Supplementary Figure S3. Size of cancer ROI in tissue specimens analyzed by MALDI-MSI. **Panel A** – correlation between the size of cancer ROI (number of spectra/image pixels) and the number of clusters generated at the first four levels of unsupervised image segmentation; the significance of the correlation was assessed using Spearman's rank correlation coefficient. **Panel B** – the size of cancer ROIs compared between the NED (No Evidence of Disease) and PD (Progressive Disease) groups. Boxplots represent minimum, maximum, lower and upper quartile, mean (black square), and median (black line); the significance of differences was assessed by the Wilcoxon rank-sum test.

Prognostic value of molecular intratumor heterogeneity in primary oral cancer and its lymph node metastases assessed by mass spectrometry imaging.

Supplementary Materials

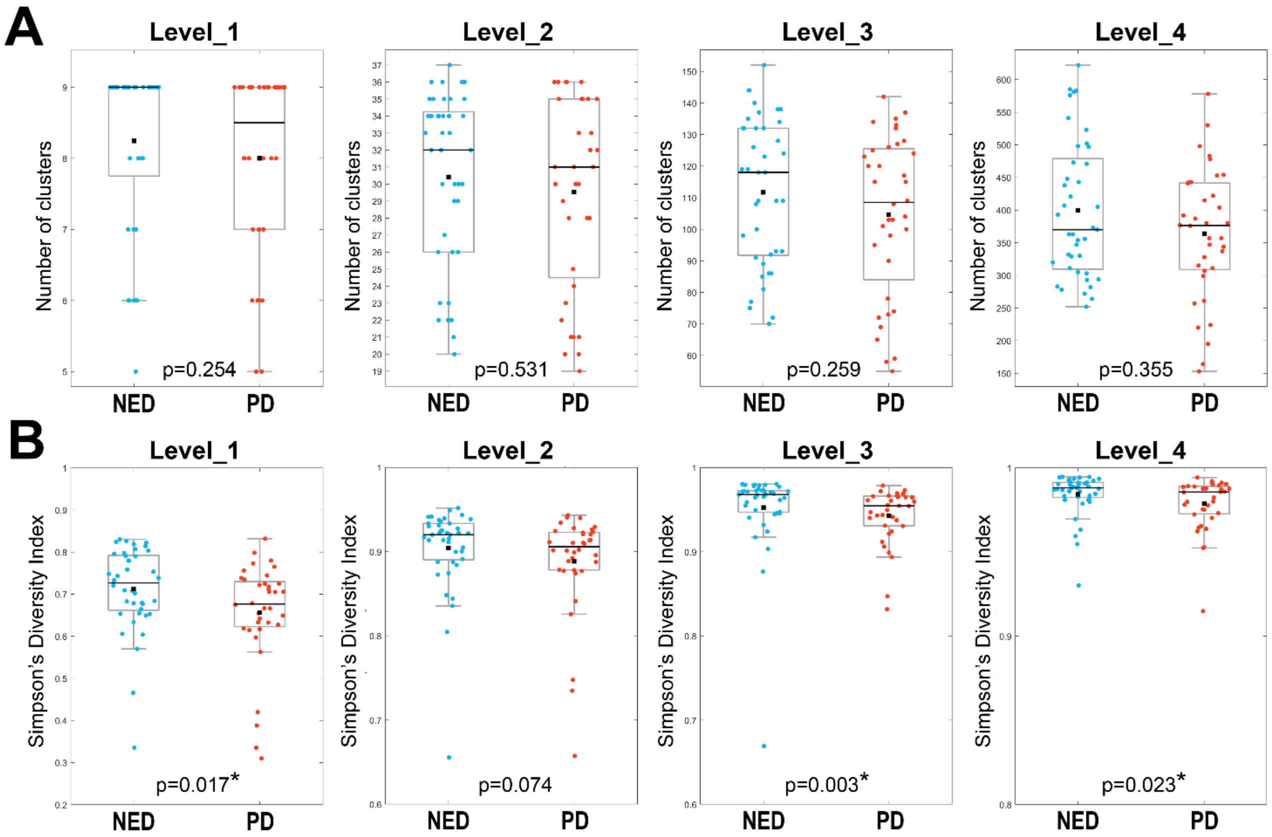

Supplementary Figure S4. Differences in the heterogeneity of cancer ROI between the NED and PD groups of patients. **Panel A** – the number of clusters generated at the first four levels of unsupervised image segmentation. **Panel B** – the Simpson's diversity index computed for the first four levels of image segmentation. Boxplots represent minimum, maximum, lower and upper quartile, mean (black square), and median (black line). The significance of differences between the NED and PD groups was assessed by the Wilcoxon rank-sum test.

Prognostic value of molecular intratumor heterogeneity in primary oral cancer and its lymph node metastases assessed by mass spectrometry imaging.

Supplementary Materials

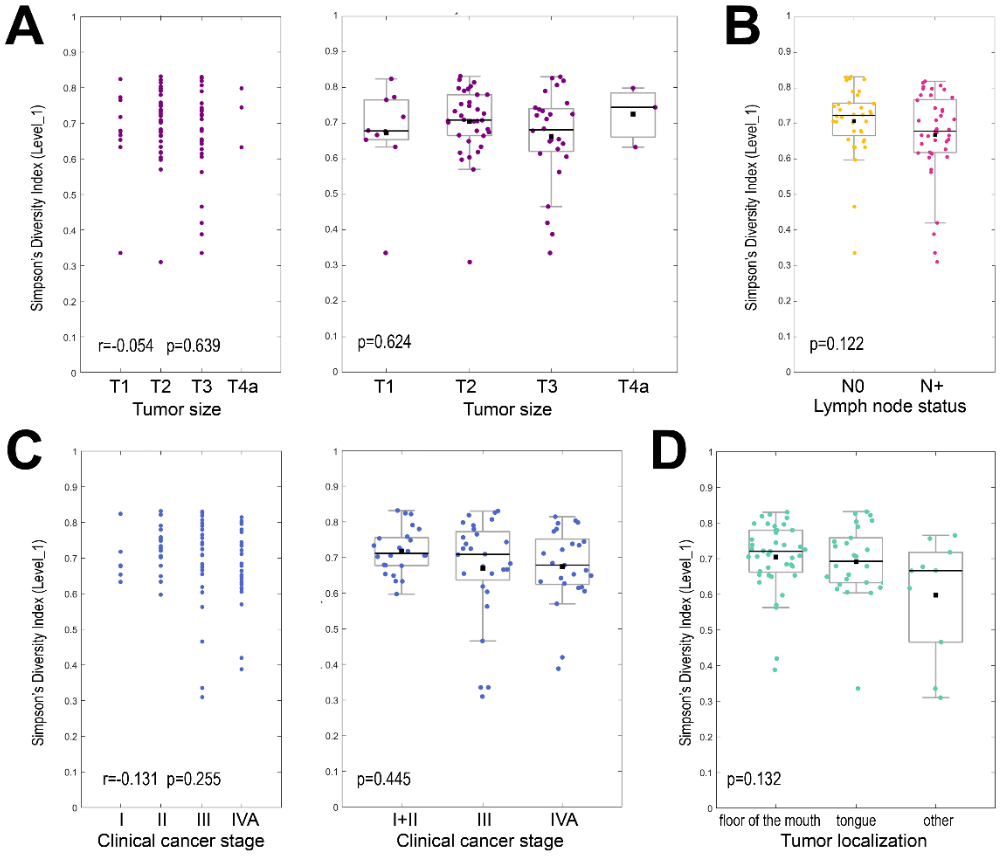

Supplementary Figure S5. Heterogeneity of cancer ROI assessed by the Simpson's diversity index at the first level of unsupervised image segmentation in different subgroups of patients. **Panel A** – comparison of patients with different primary tumor size (T): right - hypothetical correlation between the Simpson's diversity index and tumor size assessed by Spearman's rank correlation coefficient; left - the significance of differences between the subgroups assessed by the Kruskal-Wallis test. **Panel B** – comparison of patients with different regional lymph node statuses: N0 – without lymph node metastases, N+ – with synchronous lymph node metastases; the significance of differences between the subgroups assessed by the Wilcoxon rank-sum test. **Panel C** – comparison of patients with different clinical cancer stages: right - hypothetical correlation between the Simpson's diversity index and clinical cancer stage assessed by Spearman's rank correlation coefficient; left - the significance of differences between the subgroups assessed by the Kruskal-Wallis test. **Panel D** – comparison of patients with different localization of primary cancer; the significance of differences between the subgroups was assessed by the Kruskal-Wallis test. Boxplots represent minimum, maximum, lower and upper quartile, mean (black square), and median (black line).

Prognostic value of molecular intratumor heterogeneity in primary oral cancer and its lymph node metastases assessed by mass spectrometry imaging.

Supplementary Materials

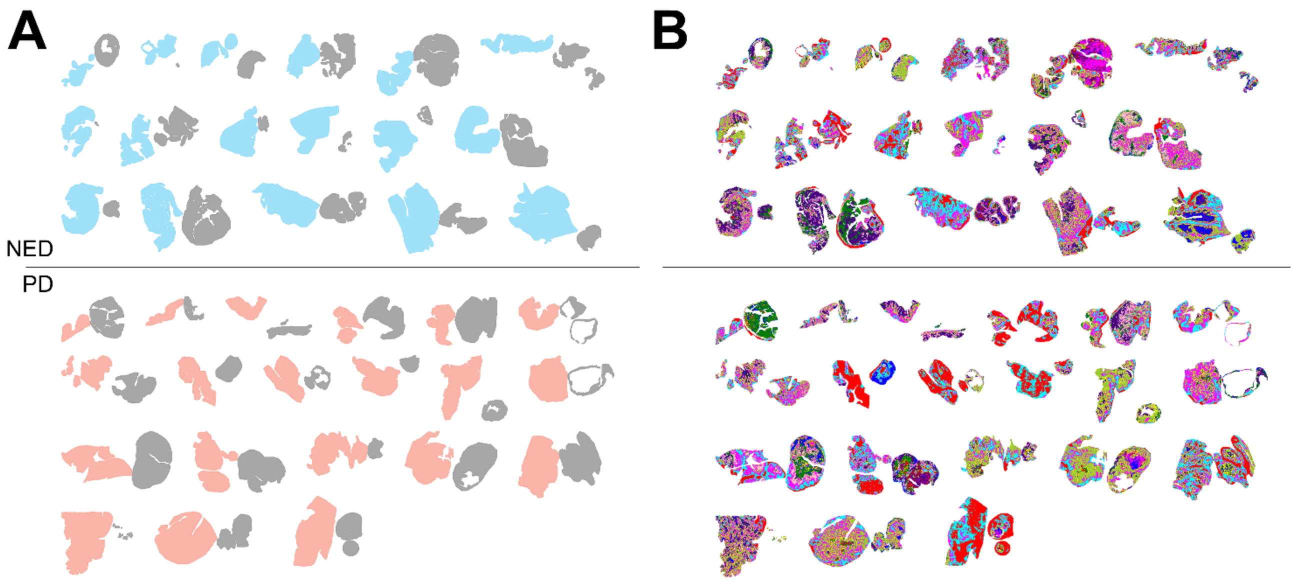

Supplementary Figure S6. The distribution of clusters identified during the unsupervised segmentation of cancer ROI in both primary tumor (T-ROI) and synchronous lymph node metastases (N-ROI). **Panel A** – schematic illustration of cancer ROIs in samples of 17 patients from the NED group and 20 patients from the PD group; T-ROIs are marked with blue and light red for NED and PD, respectively, and corresponding N-ROIs are marked with grey. **Panel B** – 16 clusters (artificially color-coded) defined at the second level of unsupervised segmentation of cancer ROIs from all 37 patients analyzed together.

Prognostic value of molecular intratumor heterogeneity in primary oral cancer and its lymph node metastases assessed by mass spectrometry imaging.

Supplementary Materials

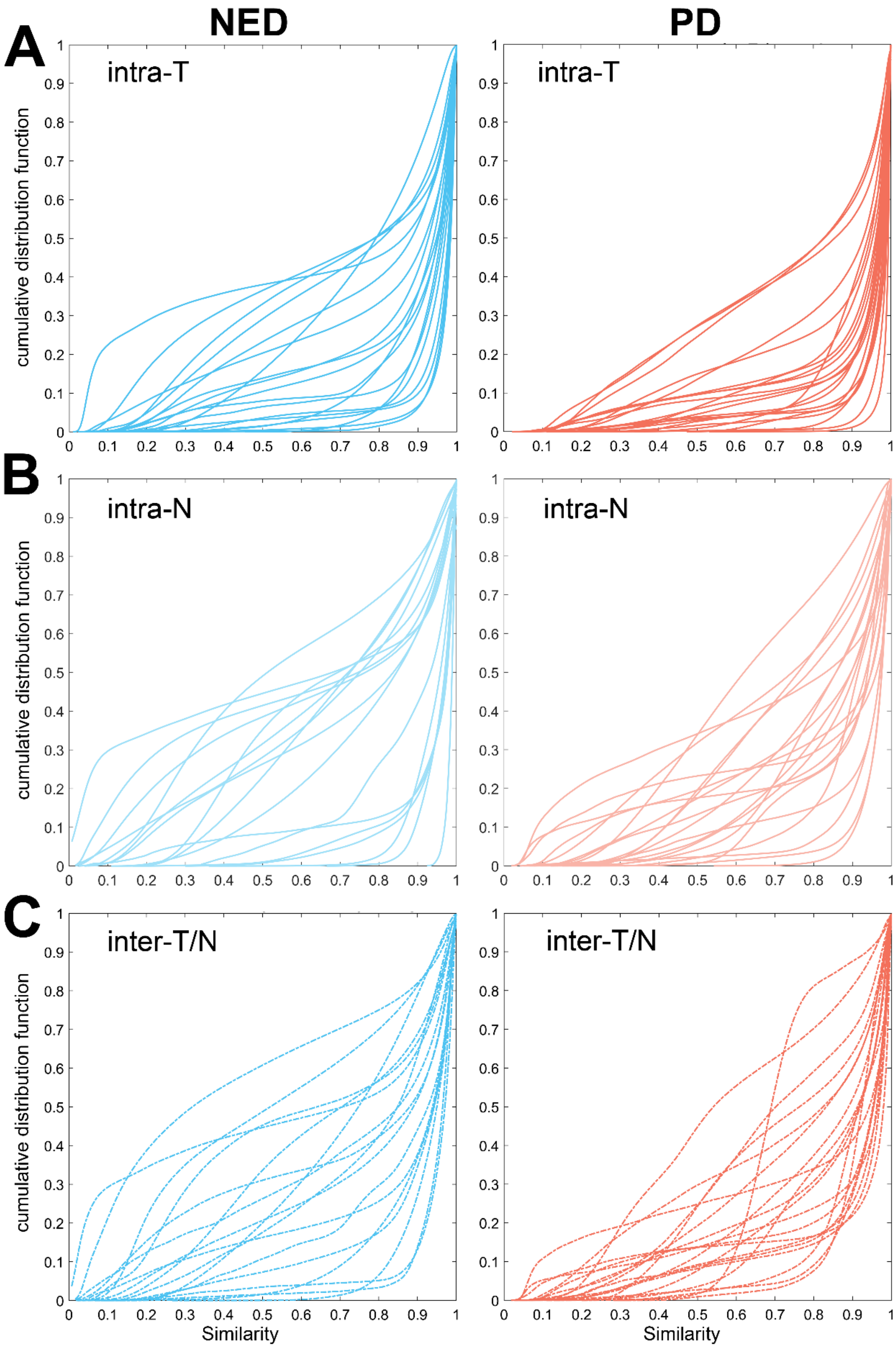

Supplementary Figure S7. The cumulative distribution function of the spectra similarity index analyzed for each patient in the NED (17 patients) and PD (20 patients) groups. **Panel A** – similarity within T-ROI areas. **Panel B** – similarity within N-ROI areas. **Panel C** – similarity between T-ROI and N-ROI areas.

Prognostic value of molecular intratumor heterogeneity in primary oral cancer and its lymph node metastases assessed by mass spectrometry imaging.

Supplementary Materials

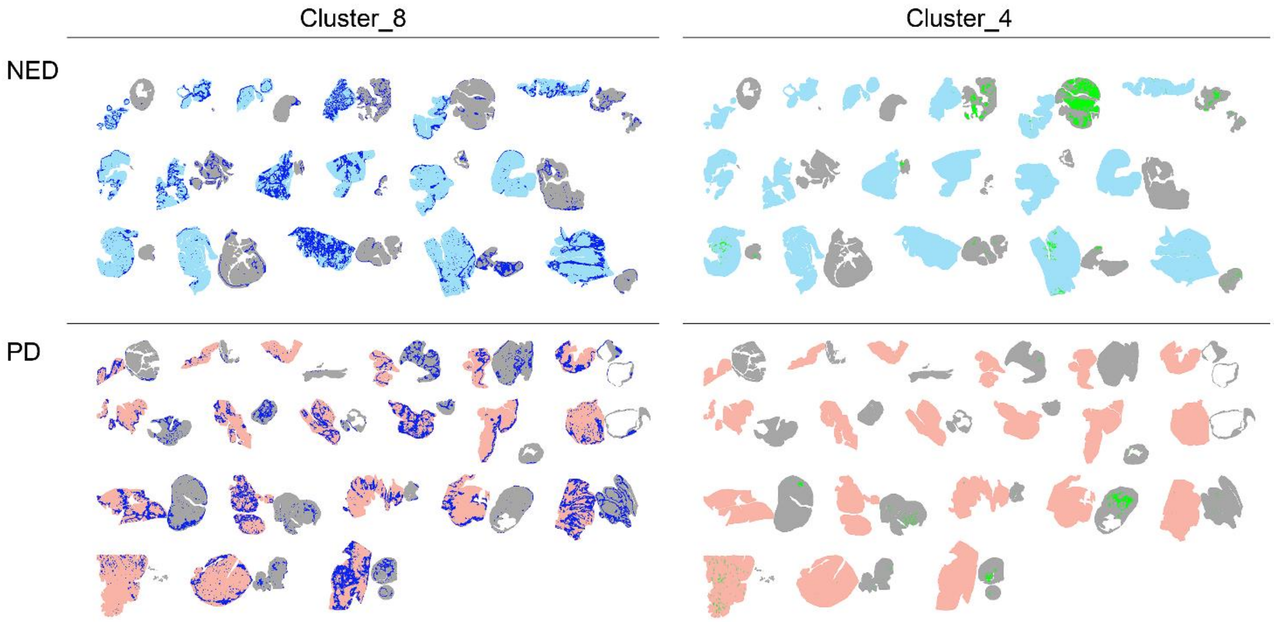

Supplementary Figure S8. The distribution of clusters identified at the second level of unsupervised segmentation of cancer ROI (both T-ROI and N-ROI) in samples of patients from the NED and PD groups. Presented are pixels that belong to Cluster_8 (left; cluster relatively overrepresented in T-ROI) or Cluster_4 (right; cluster relatively overrepresented in N-ROI).

Prognostic value of molecular intratumor heterogeneity in primary oral cancer and its lymph node metastases assessed by mass spectrometry imaging.

Supplementary Materials

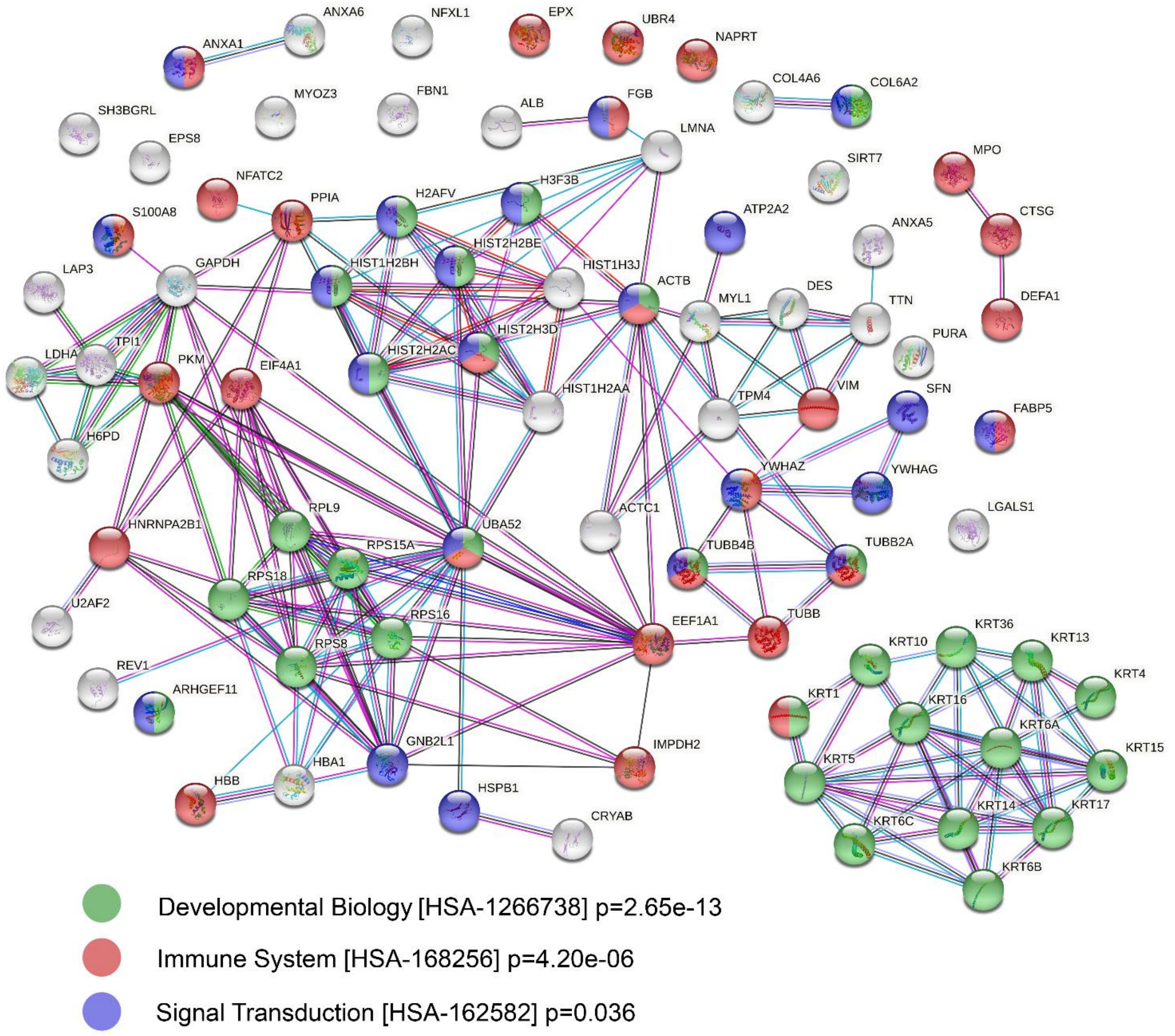

Supplementary Figure S9. The network of interactions between 87 proteins the tryptic fragments of which were putatively identified as spectral components with abundances significantly upregulated in Cluster_3# compared to other clusters at the first level of unsupervised image segmentation of all T-ROI areas; interactions between proteins and the three top over-represented Reactome pathways associated with these proteins according to an analysis performed using the STRING toolbox (shown is the corrected p-value of a pathway overrepresentation).

Prognostic value of molecular intratumor heterogeneity in primary oral cancer and its lymph node metastases assessed by mass spectrometry imaging.

Supplementary Materials

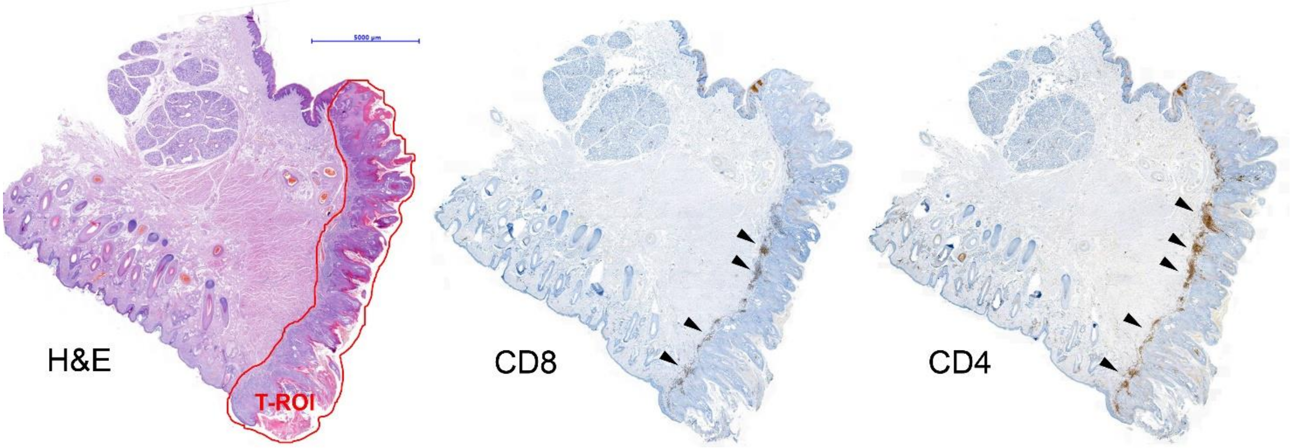

Supplementary Figure S10. Infiltration of lymphocytes at the tumor/host interface. Cancer ROI (T-ROI) was delineated at the H&E-stained tissue section (red line). Cytotoxic T cells (CD8 positive) and helper T cells (CD4 positive) were detected by immunocytochemistry in serial tissue sections (arrowheads mark clusters of T cells); illustrated is a representative specimen from a tumor with a high level of ITH assessed by MALDI-MSI and strong LHR (Lymphocytic Host Response) assessed by histopathology analysis.

Prognostic value of molecular intratumor heterogeneity in primary oral cancer and its lymph node metastases assessed by mass spectrometry imaging.

Supplementary Materials

Supplementary Protocol S1. *Preparation of tissue material for an individual patient*

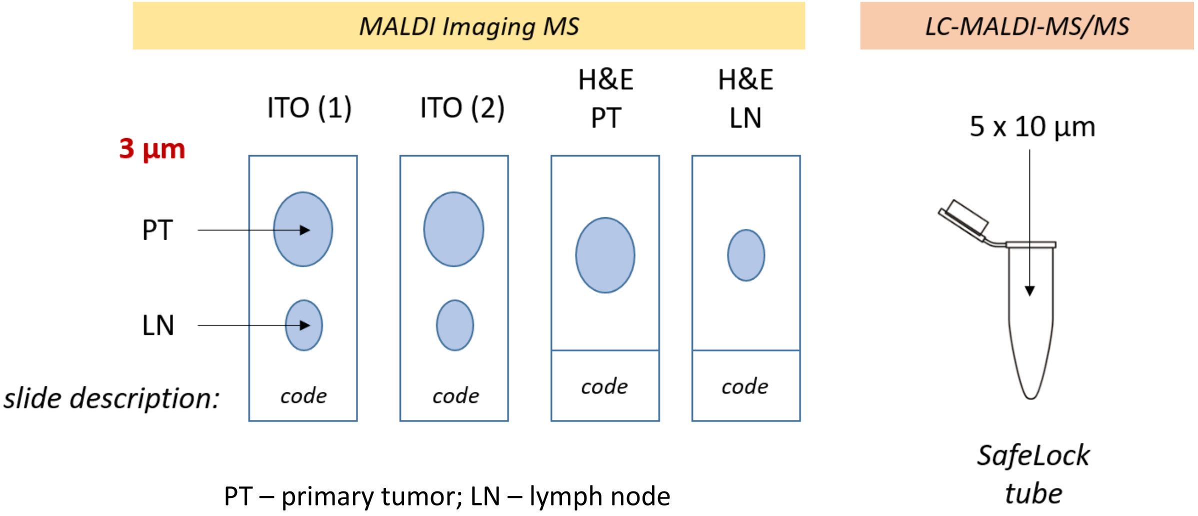

Supplementary Protocol S2. *Paraffin removal procedure*

Step	Washing solvent	Time of washing
1	100% xylene	5 min
2	100% xylene	5 min
3	99.8% ethanol	5 min
4	99.8% ethanol	5 min

Supplementary Protocol S3. *Heat-induced protein crosslinking reversal procedure*

**Instrument**	Decloaking Chamber NxGen, Biocare Medical
**Retrieval solution**	MilliQ grade ultrapure water
**Program**	110°C, 20 min
**Cooling**	on the bench: 20 min in MilliQ water
**Washing**	MilliQ water, 2 x 1 min
**Drying**	on the bench: 10 min<br>in a vacuum desiccator: 20 min

Supplementary Protocol S4a: *On-tissue trypsin digestion procedure*

**Laboratory incubator**	CLW 53 STD (Pol-Eko Aparatura)
**Humid chamber**	Sistema® KLIP IT PLUS™ food storage container (65 mm x 100 mm x 35 mm; 0.2 L) equipped with a metal plate attached to the inside of the lid, lined with a piece of laboratory filter paper (50 mm x 50 mm) on the bottom of the box
**Chamber solution**	200 µL of 100 mM NH_4HCO_3, 5% MeOH
**Slide position**	ITO glass slide (tissue section facing down) attached to the metal plate with a copper tape
**Temperature [°C]**	37
**Incubation time [h]**	18
**Drying**	in a vacuum desiccator: 30 min

Prognostic value of molecular intratumor heterogeneity in primary oral cancer and its lymph node metastases assessed by mass spectrometry imaging.

Supplementary Materials

Supplementary Protocol S4b. *SunCollect settings for trypsin and matrix deposition*

**Parameter**	**Trypsin deposition** [1)]	**Matrix deposition** [2)]
Line distance [mm]	0.5	0.5
Z [mm]	35.0	10.0
Z offset [mm]	15.0	5.0
No. layers	4	8
Flowrate 1 [µL/min]	10	10
Flowrate 2 [µL/min]	10	20
Flowrate 3 [µL/min]	10	30
Flowrate 4 [µL/min]	10	40
Speed X [mm/min]	630 (= Low (9))	630 (= Low (9))
Speed Y [mm/min]	900 (= Med (1))	900 (= Med (1))
W [µg/mm^2] [3)]	0.0063	4.13

[1)] Sequencing grade modified trypsin (Promega): 0.05 µg/µL in 10 mM NH_4HCO_3, 10% ACN

[2)] HCCA (Bruker): 5 mg/mL in 50% ACN, 0.3% TFA

[3)] Amount of deposited trypsin/matrix

Supplementary Protocol S5: *Preparation of tissue lysates for LC-MALDI-MS/MS protein identification*

1. FFPE tissue material collected in each Eppendorf tube was dewaxed with a portion of 500 µL of n-heptane (30 min incubation at room temperature), the tubes were subsequently centrifuged (20,000 RCF, 2 min), and the supernatant was replaced with a fresh portion of n-heptane (500 µL), followed by another 30-min incubation.

2. Then 25 µL of methanol was added; the tubes were centrifuged (20,000 RCF, 2 min) and supernatants were discarded. De-waxed tissue pellets were dried on air for 5 min.

3. Proteins were released from the tissue with 400 µL of tissue lysis buffer (0.1M Tris-HCl pH 8.0, 0.1M DTT, 4% SDS) upon heating at 99°C for 20 min, followed by incubation at 80°C for another 2h with constant mixing (750 rpm). The samples were then cooled down (4°C, 1 min), centrifuged at 20,000 RCF (40 min, 4°C) and supernatants were transferred to new tubes.

4. Thus obtained protein extracts were subsequently purified in a methanol/chloroform/water mixture (with sample/MeOH/$CHCl_3$/H_2O volumetric ratio of 1/4/1/3); addition of each constituent was followed with vortex mixing at maximum speed for 10 s, followed by centrifugation at 9,000 RCF for 1 min.

5. The upper layer was carefully collected and discarded, then 300 µL of MeOH was added, and the tubes were vortexed (10 s) and centrifuged at 9,000 RCF for 2 min. After that supernatants were discarded and the obtained protein pellets were washed with 1 mL of acetone (10 s of vortex mixing, centrifugation: 9,000 RCF for 2 min), supernatants were discarded and protein pellets were dried at room temperature for 5 min.

6. Pellets were then dissolved in 0.1% RapiGest SF (Waters) in 50 mM NH_4HCO_3 followed by boiling at 99°C for 10 min. Thus obtained samples were subjected to protein assay with the use of the tryptophan fluorescence method.

Prognostic value of molecular intratumor heterogeneity in primary oral cancer and its lymph node metastases assessed by mass spectrometry imaging.

Supplementary Materials

Supplementary Protocol S6: *Preparation of protein digests for LC-MALDI-MS/MS protein identification*

1. Proteins present in the lysate mixtures were subjected to disulfide bond cleavage with dithiothreitol (final concentration of DTT: 5 mM) with heating at 60°C for 30 min.
2. Alkylation was performed with iodoacetamide (final concentration of IAA: 15 mM) for 30 min in darkness (room temperature).
3. Sequencing Grade Modified Trypsin (Promega) was employed for proteolytic digestion with the enzyme to protein ratio of 1:50 (m/m). Incubation was performed for 18 h at 37°C.
4. Thus obtained digests were subsequently acidified with trifluoroacetic acid (final TFA concentration: 1%, v/v) and incubated at 37°C for 45 min. Then, the samples were centrifuged at 14,500 RCF for 20 min at room temperature and supernatants were transferred to new tubes.
5. The obtained tryptic peptides were purified with the use of TT2C18 TopTips: SpinColumn-in-a-Tip mode was employed according to the manufacturer's instructions with centrifugation at 2,150 RCF during column pre-conditioning and bed washing (both with 0.1% TFA/H_2O), and at 350 RCF during sample loading and elution (a solution of 60% ACN and 0.1% TFA was employed as an eluent).
6. Eluates were evaporated to dryness with the use of a vacuum centrifuge, then reconstituted in 20 µL of LC-MS grade water with mixing using Eppendorf Comfort Thermomixer (1000 rpm, 30 min) and subjected to peptide assay with the use of tryptophan fluorescence method.
7. Before LC-MALDI/MS/MS analysis, samples were acidified with 2 µL of 1% (v/v) trifluoroacetic acid (final TFA concentration of ca. 0.1 % TFA (v/v)).
